# Self-Assembly Behavior of Amphiphilic Janus Dendrimers in Water: A Combined Experimental and Coarse-Grained Molecular Dynamics Simulation Approach

**DOI:** 10.3390/molecules23040969

**Published:** 2018-04-21

**Authors:** Mariana E. Elizondo-García, Valeria Márquez-Miranda, Ingrid Araya-Durán, Jesús A. Valencia-Gallegos, Fernando D. González-Nilo

**Affiliations:** 1 Escuela de Ingeniería y Ciencias, Tecnológico de Monterrey, Av. Eugenio Garza Sada 2501 Sur, Monterrey 64849, Mexico; 2 Center for Bioinformatics and Integrative Biology (CBIB), Facultad de Ciencias Biológicas, Universidad Andrés Bello, Av. República 330, Santiago 8370186, Chile; valeria.marquez.m@gmail.com (V.M.-M.); ingrid.araya.duran@gmail.com (I.A.-D.); fernando.gonzalez@unab.cl (F.D.G.-N.); 3 Centro Interdisciplinario de Neurociencia de Valparaíso, Facultad de Ciencias, Universidad de Valparaíso, Gran Bretaña 1111, Playa Ancha, Valparaíso 2360102, Chile

**Keywords:** Janus dendrimers, amphiphilic, self-assembly, coarse-grained molecular dynamics

## Abstract

Amphiphilic Janus dendrimers (JDs) are repetitively branched molecules with hydrophilic and hydrophobic components that self-assemble in water to form a variety of morphologies, including vesicles analogous to liposomes with potential pharmaceutical and medical application. To date, the self-assembly of JDs has not been fully investigated thus it is important to gain insight into its mechanism and dependence on JDs’ molecular structure. In this study, the aggregation behavior in water of a second-generation bis-MPA JD was evaluated using experimental and computational methods. Dispersions of JDs in water were carried out using the thin-film hydration and ethanol injection methods. Resulting assemblies were characterized by dynamic light scattering, confocal microscopy, and atomic force microscopy. Furthermore, a coarse-grained molecular dynamics (CG-MD) simulation was performed to study the mechanism of JDs aggregation. The obtaining of assemblies in water with no interdigitated bilayers was confirmed by the experimental characterization and CG-MD simulation. Assemblies with dendrimersome characteristics were obtained using the ethanol injection method. The results of this study establish a relationship between the molecular structure of the JD and the properties of its aggregates in water. Thus, our findings could be relevant for the design of novel JDs with tailored assemblies suitable for drug delivery systems.

## 1. Introduction

Amphiphilic Janus dendrimers (JDs) are molecules composed of polar (hydrophilic) and non-polar (hydrophobic) dendritic blocks [1]. This characteristic is the key factor that favors the spontaneous self-assembly of JDs in water into complex supramolecular structures [2]. The variation in the chemical structure of JDs allows achieving a rich palette of morphologies in water, such as cubosomes, disks, tubular vesicles, helical ribbons and bilayered vesicles, termed as dendrimersomes [3].

It has been reported that characteristics of dendrimersomes make them ideal vehicles for drug delivery and as diagnostic or theranostic agents as they are monodisperse, stable up to one year in various media and can encapsulate both hydrophilic and/or hydrophobic species [4,5,6,7,8]. In addition, they exhibit mechanical properties as good as their analogous polymersomes and cholesterol stabilized liposomes [9]. Therefore, attention over these vesicles has been recently increasing.

The molecular complexity of JDs makes difficult to apply conventional geometric models for predicting their self-assembly into dendrimersomes and other structures [3]. Most of the reported studies focus on the prediction of dendrimersomes size, shape, and stability. Moreover, they are based on a single type of JDs (Percec-type) and require the full characterization of a reference structure [9,10,11]. A deep exploration of other JD structures using theoretical tools is required to understand the role of the molecular architecture in the assembly formation and molecular properties [4]. Molecular simulation can be a useful tool to gain insight into JDs self-assembly mechanism. Also, other important properties of their aggregates, difficult to evaluate via experimental methods, could be studied.

Mesoscopic molecular dynamics technologies, such as coarse-grained molecular dynamics (CG-MD), are popular alternatives to study the self-assembly of amphiphilic molecules since they reduce the computational costs when handling with large molecules and long timescales of simulation [12]. CG modeling consists of the simplification of molecular structure by mapping several atoms as single interactions sites reducing the number of degrees of freedom and maintaining the essential molecular features [13,14]. CG-MD simulation has already been applied to Percec-type JDs that formed dendrimersomes [3] and to other amphiphilic dendrimers [15].

**Figure 1:**
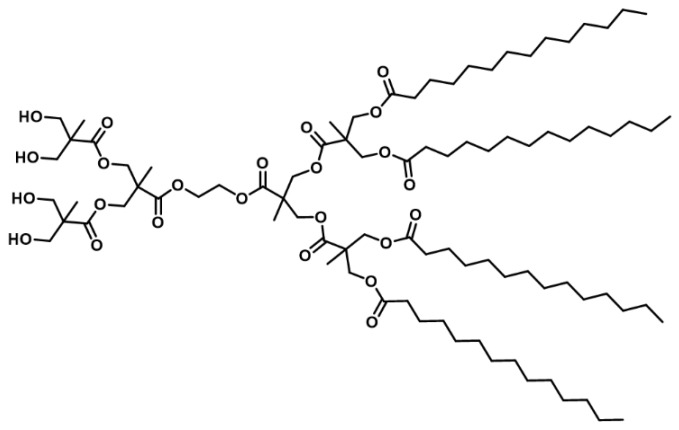
Molecular structure of the amphiphilic Janus dendrimer (JD) studied herein.

In this work, experimental methods and CG-MD simulation were combined to evaluate the self-assembly behavior in water of a second-generation bis-MPA amphiphilic Janus dendrimer (Figure 1). Bis-MPA dendrimers have already been studied [2], being perfectly suitable to build drug carriers, due to their low cytotoxicity and biodegradability. Nevertheless, this is the first report where the self-assembly phenomenon of this type of amphiphilic JD is molecularly explored using a CG-MD simulation. Results obtained by CG-MD simulation agreed with the information collected in the experimental method: both strategies coincide in the formation of assemblies in water, with no interdigitated bilayer membranes. However, differences in particle size and morphology of these assemblies were found. Although these differences, the CG-MD simulation results provide important insights related to the mechanism of formation of the assemblies and dendrimer parameters that are difficult to obtain through experimental methods. This information is relevant for the prediction of supramolecular structure properties of novel JDs with application in the development of drug delivery systems.

## 2. Results

### 2.1. Formation and Characterization of Assemblies by Experimental Methods

#### 2.1.1. Giant Assemblies

**Figure 2:**
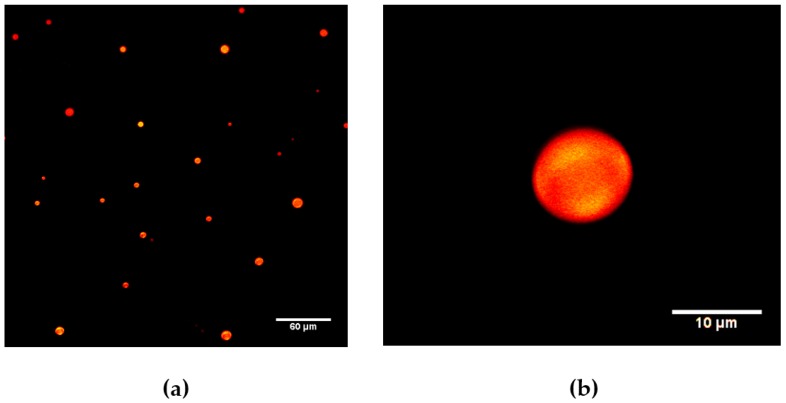
(**a**) Confocal microscopy images of JDs assemblies formed by the thin-film hydration method and encapsulating Nile red (hydrophobic dye); (**b**) Optical section of an individual assembly taken at 9.9 µm from the beginning of the structure.

Confocal microscopy of the assemblies produced via thin-film hydration method showed the formation of spherical structures, with diameters less than 20 µm (Figure 2a). With the thin-film hydration method, a low production of assemblies and the incomplete dispersion of the JDs film in water were observed. The JDs were able to encapsulate the hydrophobic dye Nile Red as confirmed by confocal microscopy. Optical sections of assemblies showed the accumulation of the dye within the whole structure of the assemblies. No presence of water inside the structures was observed. Figure 2b shows an optical section of an assembly, taken at 9.9 µm from the beginning of the structure. The results of dye encapsulation experiments indicate that hydrophobic domains mainly compose the structures, discarding the presence of dendrimersomes. Assemblies could be formed by multiple layers of dendrimers.

#### 2.1.2. Small Assemblies

Assemblies obtained via ethanol injection method were analyzed using dynamic light scattering (DLS). This method of production generated assemblies homogeneous in size, with a Z-average of 69.6 ± 1.92 nm (diameter) and PDI of 0.14 ± 0.01. In addition, the assemblies presented a negative ζ-potential of −30.1 ± 1.72 mV. The assemblies did not show significant differences in size after 20 days at 25 °C (*p*-value > 0.05, Figure S1) and were stable in a temperature range of 25–70 °C (Figure S2).

**Figure 3:**
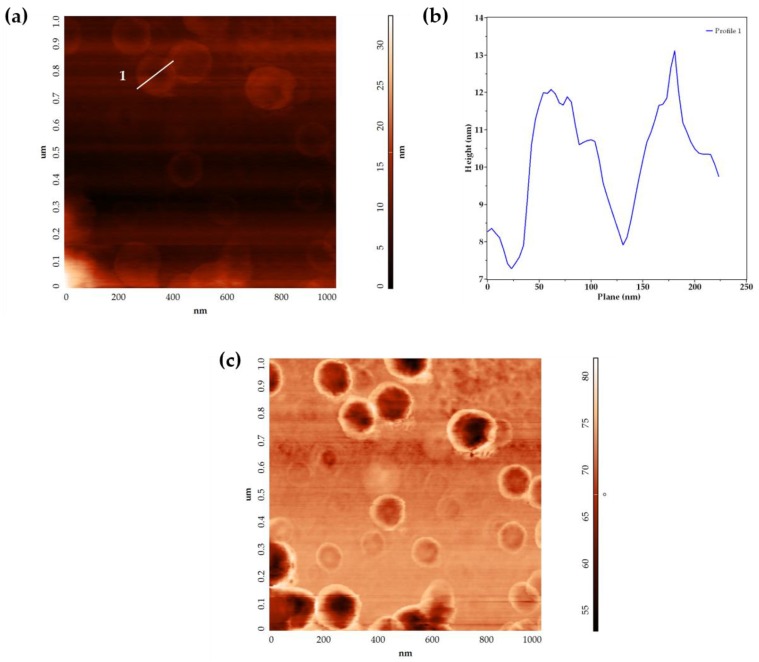
Atomic force microscopy (AFM) images of air-dried small assemblies obtained using ethanol injection method. (**a**) AFM height image and (**b**) cross-section profile determined from line 1; (**c**) AFM phase image.

Assemblies were also analyzed by atomic force microscopy (AFM), which allowed the observation of isolated structures with concave and convex morphologies in the AFM height images (Figure 3a). Profile analysis of an AFM height image is presented in Figure 3b. Convex structures with larger dimensions product of assemblies’ fusion, were observed (data not shown). All structures presented larger diameters (144 ± 21.6 nm) than related heights (4.30 ± 0.91 nm). The diameter values were higher than those calculated by DLS. The AFM phase images (Figure 3c) showed a dark area in the center of the assemblies (negative phase shift) and a bright area in their contour (positive phase shift), indicating differences in the surface properties of the samples.

### 2.2. Coarse-Grained Molecular Dynamic Simulation

**Figure 4:**
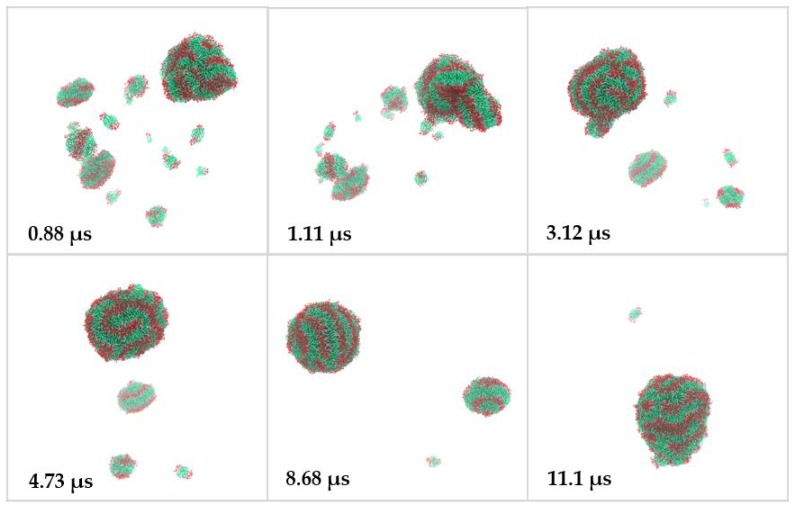
Representative images of the self-assembly process of JDs in water at different stages of the coarse-grained molecular dynamics (CG-MD) simulation. Color code: polar head group, red; hydrophobic tails, green; and hydrophilic groups, gray. Water is not shown.

**Figure 5:**
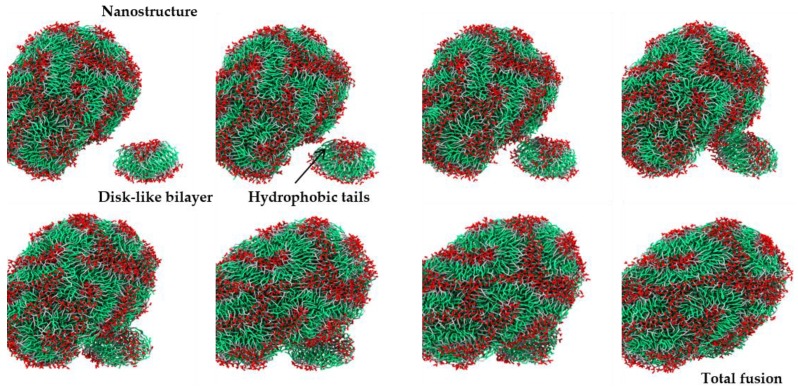
Snapshot of fusion mechanism of a disk-like bilayer and a major size nanostructure. Color code: polar head group, red; hydrophobic tails, green; and hydrophilic groups, gray. Water is not shown.

**Figure 6:**
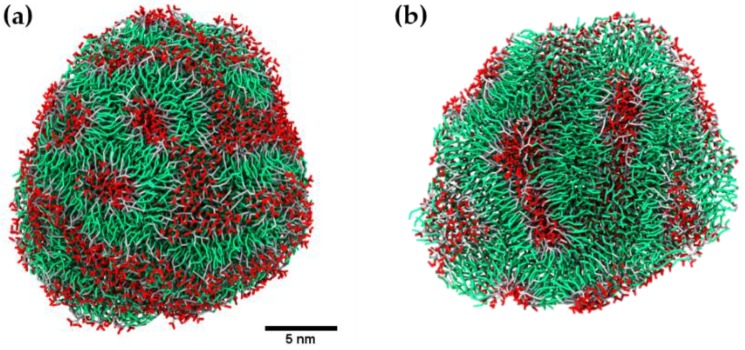
(**a**) Final assembly obtained by coarse-grained molecular dynamics (CG-MD) simulation. (**b**) A cross-sectional view of the nanostructure. Time of simulation: 13 µs. Color code: polar head group, red; hydrophobic tails, green; and hydrophilic groups, gray. Water is not shown.

The self-assembly of dendrimers in water at different stages of the CG-MD simulation is shown in Figure 4. At the beginning of the simulation, the randomly distributed dendrimers self-assembled into clusters, including small disk-like bilayers with a non-zero spontaneous curvature. More complex clusters, formed by several stacked bilayers, were also identified at the early stages of the simulation. Along with simulation, the small clusters continued aggregating to large clusters through hydrophobic interactions. Figure 5 presents images taken from the simulation, in which a fusion event between a nanostructure and a disk-like bilayer is observed. At the end of the simulation, the majority of the bilayered assemblies clustered together. The reaching of the equilibrium stage of the system was confirmed by the calculation of solvent accessible surface area (SASA) of the dendrimers during the simulation (Figure S3). After 10 μs of simulation, this parameter remained constant. At the 13 μs of simulation, a quasi-spherical nanostructure with an average volume of 12.9 nm^3^, diameter around 20 nm and aggregation number of 1689 JDs, was obtained (Figure 6a). This final structure is constituted by entangled dendrimer bilayers, with hydrophobic sites exposed to aqueous media and does not present dendrimersome morphology as seen in the cross-sectional view of the final structure (Figure 6b).

**Figure 7:**
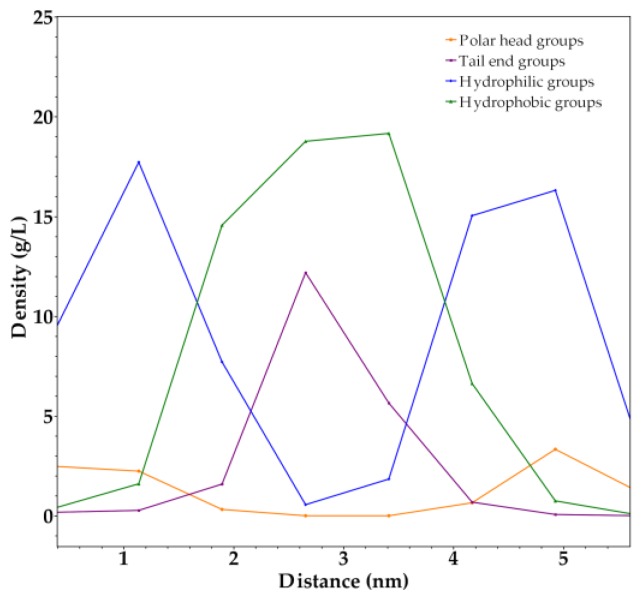
Density profile analysis of a selected patch from the final nanostructure. Size of selected patch considered: 6.5 × 5.4 × 7.4 nm^3^.

A patch was selected from the final simulation nanostructure. The density profile analysis of the components of this patch (Figure 7) displays the decrease of polar head groups and total hydrophilic groups density, as the measured distance approaches the center of the selection. On the contrary, the density of tail end groups and total hydrophobic groups increased in this zone and decreases as the measured distance moves away from the center of the selection. This data confirms that dendrimers tend to arrange into bilayers that are forming the final nanostructure.

A bilayer thickness of 4.1 nm with a hydrophobic part length of 1.8 nm was determined from the selected patch. In addition, a dendrimer length of 2.0 nm with a hydrophobic tail length of 0.7 nm was determined. It is important to note that bilayer thickness and its hydrophobic part length were more than twice as large as the dendrimer and its hydrophobic tail length, respectively. Hence, there is no interdigitation among bilayer components.

## 3. Discussion

In this study, experimental and computational methods were applied to evaluate the self-assembly behavior in water of a second-generation bis-MPA amphiphilic Janus dendrimer.

Spherical giant assemblies with diameters less than 20 µm were produced using the thin-film hydration method. Unlike previous reports, where giant unilamellar dendrimersomes have been formed by the gentle hydration (natural swelling) of JD films [3,5,6,7,16], the giant assemblies obtained herein did not present this vesicle-like morphology. Instead, aggregates of dendrimers without water entrapped inside were obtained. No formation of dendrimersomes can be attributed to the poorly hydration of the dendrimer film caused by the insufficient electrostatic repulsion between the adjacent lamellae, as seen in neutral phospholipids [17,18]. Moreover, the preparation methodology adopted uses heat to promote the lamellae hydration. According to Ianiro et al. [19], this condition causes the loss of the hydrogen bonds between water and hydrophilic domains, reducing the overall solubility of the amphiphile. It is necessary to explore other preparation procedures, varying the temperature of thin-films rehydration or using other production techniques for giant vesicles, such as microfluidics. 

Small assemblies, generated via ethanol injection, presented characteristics that indicate the obtaining of dendrimersomes. In AFM height images, concave structures with diameters larger than related heights were mainly observed. This morphology has been previously reported in dried liposomes and it has been attributed to a structural collapse due to the evaporation of water from vesicle interior and the adsorption on the substrate [20,21]. This behavior was also observed in dendrimersomes, as reported by Giustini et al. [22]. Considering the apparent height of the higher outline of the structures (4.30 ± 0.91 nm) that compares with the typical dendrimersome bilayer thickness (5 to 8 nm) [2,22,23], it can be deduced that this value corresponds to the membrane thickness of the assemblies. The collapsed structure of the assemblies was confirmed by the AFM phase images, where a phase shift was observed. Ruozi et al. [20] reported that this variation could be affected by the hydration degree of the bilayer and consequently by the local surface properties of vesicles. The negative phase shift, identified in the depressed central portion of the structure, can be related to a flattened layer of lipids with high viscosity. On the other hand, the positive phase shift observed in the higher outline of the structure suggests that the lipids are still hydrated with a relatively low viscosity environment [20,24]. Although AFM images allowed the identification of dendrimersomes, one limitation of this study is that assemblies were analyzed as dried samples. Other techniques, including environmental scanning electron microscopy (ESEM) [20,25] and liquid-cell transmission electron microscopy (LCTEM) [26], can be used for the visualization of small assemblies in the hydrated state. Nonetheless, unlike AFM, the resolution of ESEM do not provide detailed information related to the surface and architecture of the small vesicles [20], and LCTEM has to be improved and it may not be accessible to researchers due to its short time development [27].

In the CG-MD simulation, the mechanism of final assembly formation involves the agglomeration of dendrimers into disk-like rippled bilayers that subsequently merged forming a larger multilayered nanostructure. Reported mechanisms, using computer simulation and macromolecules different to dendrimers (polymers [28,29], cationic-anionic surfactants [12], and phospholipids [30,31]) describe three stages in this process: the nucleating, fusion and curling stages. In the nucleating stage, small clusters of amphiphilic molecules are formed; during the fusion stage, small micelles fuse to form disk-like aggregates; and finally, in the curling stage these disk-like aggregates curl up and close to form vesicles [12]. In contrast, JDs presented aggregation into bilayers rather than micelles, this behavior could be due to the lack of molecules that being able to form the lateral edges of the micelles. The geometrical condition in the molecular structure of the amphiphiles to form that edges in micelles is that their packing parameter factor accomplish *v*/*a_o_l_c_* < 1/3, where *v* is the volume of their hydrocarbon chains, *a_o_* is their optimal area per head group that minimize the free energy and *l_c_* is roughly the length of the hydrocarbon chain [32]. According to this, the volume of the JDs hydrocarbon chains is too high compared to the polar head group area, causing that they cannot form micelles, in contrast to single-tailed amphiphilic dendrimers, such as PAMAM-G2 derivatives [15].

In accordance with our results, Percec et al. [3] reported differences in resulting structures of CG-MD simulation of an uncharged amphiphilic JD, dependent on the concentration of the initially dispersed molecules. Also, Arai et al. [33] reported this behavior with lipid membrane solutions simulations, where they observed network and sponge morphologies, closer to our results, at higher lipid concentrations. Furthermore, using experimental methods, Fedeli et al. [2] reported the obtaining of aggregates, homogeneous in dimensions and with diameters around 50 nm (observed in TEM images) from a bis-MPA based second-generation JD with a molecular structure like the JD simulated herein. According to Fedeli et al., these aggregates cannot be explained as formed by individual bilayers and they proposed that the obtained nanospheres are formed from bent and rolled-up bilayer structures. On the other hand, the authors describe the formation of vesicles from higher generation JDs (hydrophobic-hydrophilic block generations: G2-G3 and G3-G3). According to this result and previous studies of CG-MD focused on the self-assembly of different generation amphiphilic dendrimers [15], generation of the hydrophilic block influences the morphology of the assemblies and higher dendrimer generation could derive in the formation of vesicles. One limitation of our study is that we do not evaluate different concentrations or compositions of dendrimers in the simulation, to explore other assemblies’ morphologies. Nevertheless, to our knowledge, this is the first report where the self-assembly phenomenon of this type of bis-MPA amphiphilic JD is molecularly explored using a CG-MD simulation.

It is important to note that, in the CG-MD simulation, the bilayer thickness and its hydrophobic part length were more than twice as large as the dendrimer and its hydrophobic tail length, respectively. Moreover, comparing this value with the bilayer thickness deducted from AFM height images of small assemblies, a non-significant difference was found (*p*-value > 0.05). This information indicates that there is no interdigitation among bilayer components. Interdigitation degree in bilayers is related to the size, hardness, and stability of aggregates [9]. This parameter is influenced by the length of hydrophobic tails [15] and other factors, such as the formation of hydrogen bonds between the hydrophilic segments of the membrane dendrimers, which prevent the interdigitation [11]. The dendrimer studied here is composed of bis-MPA moieties with hydroxyl groups that form this type of interactions and may be influencing the arrangement of the dendrimers in the bilayers.

CG-MD simulation did not represent in totality the results obtained by the experimental methods. In comparison with small assemblies obtained by ethanol injection method, CG-MD simulation assembly presented smaller particle size and different morphology. These differences may be due to experimental variables involved in the method applied for assemblies’ formation, such as: stirring speed, injection flow and ethanol concentration [34,35]. These variables are unable to be reproduced by simulation and have an impact on the properties of the assemblies. Furthermore, the initial concentration of the dendrimer and spatial disposition in the simulated system may also influence the morphology of the assemblies. However, giant assemblies presented similar morphology to the CG-MD simulation assembly. In this case, the process of assemblies’ formation through hydration of JDs lamellas is influenced by the interaction between hydrophilic sections of the JDs, as described above. In like manner, simulation environment with a high concentration of JD favors the interaction between polar heads of the dendrimers, preventing the formation of disk-like aggregates with larger dimensions and with the ability to curl up to form a vesicle.

The findings of this work established a relationship between the molecular structure of the amphiphilic JD and the characteristics of their assemblies in water. Likewise, the developed CG model could be adapted to specific structural modifications of the dendrimer, to study in depth this relationship. Thus, this is relevant for the design of novel, tailored JD assemblies.

Further studies should explore systems with different concentrations and composition of dendrimers and/or use pre-assembled bilayer structures as a starting point for the CG-MD simulation to get more information about the dendrimersome formation process. Also, the final state of the CG-MD simulation can be transformed to full atomistic simulation, to study in more detail the dendrimer-dendrimer interactions. Another subject to be explored is the dendrimer structure modifications that favor the interdigitation degree and thus stability of the assemblies.

## 4. Materials and Methods

### 4.1. General Information

All chemicals were purchased from Sigma-Aldrich (St. Louis, MO, USA) and used without any further purification except where noted otherwise. Ethyl acetate, dichloromethane, and hexane (all reagent grade) were purchased from CTR scientific (Monterrey, Mexico) and distilled prior to use. Ultrapure water (18.2 MΩ.cm) was obtained from a Milli-Q system from Millipore (Billerica, MA, USA). The catalyst 4-(dimethylamino)pyridinium *p*-toluenesulfonate (DPTS) was synthesized according to Moore et al. [36]. Isopropylidene-[G-2]-benzyl ester and its deprotected product OH-[G-2]-benzyl ester were obtained according to the procedure reported by Appel et al. [37]. Isopropylidene-[G-2]-COOH was obtained from isopropylidene-[G-2]-benzyl ester hydrogenolysis, following the procedure reported by Ihre et al. [38]. Preparative flash column chromatographies were carried out using silica-gel with a particle size of 40–63 μm (SiliaFlash^®^ P60, SiliCycle, Quebec city, QC, Canada). Analytical thin layer chromatographies (TLC) were performed on silica gel plastic plates (TLC Silica gel 60 F_254_, Merck, Darmstadt, Germany).

### 4.2. Instruments for Dendrimers Characterization

#### 4.2.1. Nuclear Magnetic Resonance (NMR)

^1^H- and ^13^C-NMR spectra were recorded at 500.13 and 125.76 MHz, respectively, on a Bruker Advance III spectrometer (Billerica, MA, USA), using *d*-chloroform (CDCl_3_) as a solvent. The solvent signal was used as an internal standard.

#### 4.2.2. Mass Spectra

Mass spectra were obtained using an Autoflex II MALDI-TOF mass spectrometer (Bruker Daltonics, Bremen, Germany). Measures were performed in linear positive mode, using a nitrogen laser (337 μm) at 50 Hz frequency. The acceleration voltage was 19.50 kV, with delay time acquisition. The analytical samples were obtained by the dry-droplet method. Briefly, 1 μL of an analyte solution in methanol (1 mg/mL) was loaded on the MALDI plate (MTP 384 target plate polished steel BC, Bruker Daltonics, Bremen, Germany) and allowed to dry at 23 °C. Each sample was covered with 2 μL of matrix (α-cyano-4-hydroxycinnamic acid) solution (10 mg/mL, 50% acetonitrile, water 47.5% and 2.5% trifluoroacetic acid) and allowed to dry at 23 °C before the plate was inserted into the vacuum chamber of the MALDI instrument. Data analysis was carried in FlexAnalysis 3.0 software (Bruker Daltonics, Bremen, Germany).

### 4.3. Synthesis and Characterization of Amphiphilic Janus Dendrimers

In general, the growth of dendrimer was performed via Steglich esterifications, which involve the use of *N*,*N*′-dicyclohexylcarbodiimide (DCC) and DPTS as activating agents. The reaction scheme is presented in Figure S4.

#### 4.3.1. Dendron **1** and General Esterification Procedure

Isopropylidene-[G-2]-benzyl ester (0.409 g, 0.896 mmol), myristic acid (1 g, 4.38 mmol) and DPTS (0.113 g, 0.384 mmol) were dissolved in dichloromethane (5 mL). To this, a solution of *N*,*N*′-dicyclohexylcarbodiimide (DCC, 1.042 g, 5.05 mmol) in dichloromethane (3 mL) was added dropwise. The reaction mixture was stirred for 24 h at 23 °C. Once the reaction was complete the white precipitate (*N*,*N*′-dicyclohexylurea, DCU) was filtered off using a glass filter and washed with dichloromethane (5 mL). The organic solvent was removed with a rotary evaporator. The crude product was precipitated in ethanol (68 mL) at 4 °C. The compound was obtained as a white solid: 0.900 g (79.1%). ^1^H-NMR (500 MHz, CDCl_3_) δ 7.45–7.28 (m, ~5H, -CH=), 5.15 (s, 2H, -O-CO-CH_2_-C-), 4.30–4.23 (m, 4H, -CHH′-O-CO-[G-1] non-polar block), 4.18–4.10 (m, ~8H, -CHH′-O-CO-[G-2] non-polar block), 2.27 (t, *J* = 7.6 Hz, 8H, -O-CO-CH_2_-CH_2_-(CH_2_)_10_-), 1.64–1.42 (m, 8H, -O-CO-CH_2_-CH_2_-(CH_2_)_10_-), 1.35–1.20 (m, ~83H, -C-CH_3_ and -O-CO-CH_2_-CH_2_-(CH_2_)_10_-), 1.16 (s, 6H, -C-CH_3_), 0.88 (t, *J* = 6.9 Hz, 12H, -(CH_2_)_10_-CH_3_). ^13^C-NMR (126 MHz, CDCl_3_) δ 173.17, 172.10, 172.00, 135.42, 128.69, 128.50, 128.36, 67.13, 65.76, 65.03, 46.75, 46.44, 34.17, 34.06, 31.94, 29.71, 29.68, 29.67, 29.65, 29.51, 29.37, 29.31, 29.16, 24.89, 22.70, 17.72, 17.58, 14.11. MALDI-TOF MS (*m*/*z*) [M-H + 2Na]^+^ calcd. for C_78_H_135_Na_2_O_14_: 1342.88; found 1342.29.

#### 4.3.2. Dendron **2**

Pd/C (10%, 0.29 g), was added to a solution of dendron **1** (2.88 g, 2.22 mmol) in a mixture of ethyl acetate and dichloromethane (5:1, 30 mL). The apparatus for catalytic hydrogenolysis was evacuated of air and filled with H_2_ (40 psi). After 5 h of shaking at 23 °C, the reaction was complete. The catalyst was filtered off and carefully washed with ethyl acetate (5 mL). The solvent of the filtrate was eliminated with a rotary evaporator to give the compound as a white solid: 2.65 g (99%). This product was used without further purification. ^1^H-NMR (500 MHz, CDCl_3_) δ 4.30–4.15 (m, 12H, -CHH′-O-CO-[G-1] non-polar block, -CHH′-O-CO-[G-2] non-polar block), 2.29 (t, *J* = 7.6 Hz, 8H, -O-CO-CH_2_-CH_2_-(CH_2_)_10_-), 1.66–1.52 (m, 8H, -O-CO-CH_2_-CH_2_-(CH_2_)_10_-), 1.35–1.17 (m, 89H, -C-CH_3_ and -O-CO-CH_2_-CH_2_-(CH_2_)_10_-), 0.88 (t, *J* = 6.9 Hz, 12H, -(CH_2_)_10_-CH_3_).^13^C-NMR (126 MHz, CDCl_3_) δ 175.33, 173.33, 173.31, 172.04, 65.67, 65.14, 65.11, 46.47, 46.35, 34.08, 31.94, 29.71, 29.69, 29.67, 29.66, 29.52, 29.37, 29.31, 29.17, 24.89, 22.70, 17.77, 17.54, 14.11. MALDI-TOF MS (*m*/*z*) [M-H + 2Na]^+^ calcd. for C_71_H_129_Na_2_O_14_: 1252.76; found 1251.96.

#### 4.3.3. Dendron **3**

Dendron **2** (1.022 g, 0.846 mmol), ethylene glycol (0.269 g, 4.33 mmol), DPTS (0.049 g, 0.166 mmol), and DCC (0.209 g, 1.01 mmol) were allowed to react for 24 h in CH_2_Cl_2_ (12 mL) following the general esterification procedure. The crude product was purified by flash column chromatography using a mixture of 20:80 ethyl acetate/hexane, increasing to 100% ethyl acetate, to give compound 3A as a white solid: 0.781 g (75.3%). ^1^H-NMR (500 MHz, CDCl_3_) δ 4.40–4.08 (m, 14H, -CHH′-O-CO-[G-1] non-polar block, -CHH′-O-CO-[G-2] non-polar block and -CO-O-CH_2_-CH_2_-OH), 3.89–3.76 (m, 2H, -CO-O-CH_2_-CH_2_-OH), 2.29 (t, *J* = 7.6 Hz, 8H, -O-CO-CH_2_-CH_2_-(CH_2_)_10_-), 1.62–1.54 (m, 8H, -O-CO-CH_2_-CH_2_-(CH_2_)_10_-), 1.34–1.20 (m, 89H, -C-CH_3_ and -O-CO-CH_2_-CH_2_-(CH_2_)_10_-), 0.88 (t, *J* = 6.8, 12H, -(CH_2_)_10_-CH_3_). ^13^C-NMR (126 MHz, CDCl_3_) δ 173.32, 172.46, 172.18, 67.09, 65.92, 65.04, 60.77, 46.77, 46.47, 34.05, 33.93, 31.91, 29.68, 29.65, 29.62, 29.48, 29.34, 29.27, 29.13, 24.85, 22.67, 17.77, 17.61, 14.08. MALDI-TOF MS (*m*/*z*) [M-H + 2Na]^+^ calcd. for C_73_H_133_Na_2_O_15_: 1296.81; found: 1296.75.

#### 4.3.4. Amphiphilic Janus Dendrimer (JD)

Dendron **3** (0.781 g, 0.624 mmol), isopropylidene-[G-2]-COOH (0.563 g, 1.26 mmol), DPTS (0.076 g, 0.258 mmol), and DCC (0.335 g, 1.62 mmol) were allowed to react for 24 h in CH_2_Cl_2_ (18 mL) following the general esterification procedure. The crude product was purified by flash column chromatography using a mixture of 30:70 ethyl acetate/hexane, to give a white solid: 0.734 g (81%). Deprotected dendrimer was obtained following the procedure described by Tuutila et al. [39]. Briefly, the purified solid (0.210 g, 0.124 mmol) was solubilized in CH_2_Cl_2_ (5 mL) and diluted with methanol (5 mL). One teaspoon of Dowex^®^ 50WX8 resin was added, and the reaction mixture was stirred at 55 °C for 24 h. After this time, the resin was filtered off and washed with dichloromethane (5 mL). The organic solvent was removed with a rotary evaporator to give the compound 4A as a white solid: 0.156 g (78%). ^1^H-NMR (500 MHz, CDCl_3_) 4.43 (d, *J* = 11.1 Hz, 2H, -CHH′-O-[G-1] polar block), 4.39–4.09 (m, 18H, -O-CO-CH_2_-CH_2_-O-CO-, -CHH′-O-[G-1] polar block, -CHH′-O-[G-1] non-polar block and -CHH′-O-CO-[G-2] non-polar block), 3.87–3.77 (m, 4H, -CHH′-OH), 3.76–3.64 (m, 4H, -CHH′-OH), 2.29 (t, *J* = 7.6 Hz, 8H, -O-CO-CH_2_-CH_2_-(CH_2_)_10_-), 1.64–1.51 (m, 8H, -O-CO-CH_2_-CH_2_-(CH_2_)_10_-), 1.34–1.20 (m, 92H, -C-CH_3_ and -O-CO-CH_2_-CH_2_-(CH_2_)_10_-), 1.06 (s, 6H, -C-CH_3_), 0.88 (t, *J* = 6.9 Hz, 12H, -(CH_2_)_10_-CH_3_). The ^1^H-NMR spectrum and peak assignments are presented in Figure S5. ^13^C-NMR (126 MHz, CDCl_3_) δ 175.18, 173.42, 173.41, 172.79, 172.27, 172.08, 68.25, 68.19, 66.09, 65.78, 65.22, 65.17, 65.00, 62.99, 62.95, 49.94, 46.87, 46.70, 46.66, 46.62, 34.20, 32.07, 29.84, 29.81, 29.80, 29.78, 29.65, 29.50, 29.44, 29.42, 29.30, 25.02, 22.82, 18.13, 17.92, 17.77, 17.63, 17.25, 14.23. MALDI-TOF MS (*m*/*z*) [M + 2Na]^+^ calcd. for C_88_H_158_Na_2_O_24_: 1646.16; found 1648.92.

### 4.4. Formation and Characterization of Assemblies (Experimental Method)

To assess different properties of aggregation behavior in water, two sizes of assemblies were created through different methods. Giant assemblies (size ≥ 1 μm) were used to identify hydrophobic and hydrophilic domains in the assemblies. While small assemblies (size ≤ 100 nm) were produced to evaluate size, polydispersity index (PDI), ζ-potential and morphology of the assemblies. Aqueous dispersions of JDs were carried out using the liposome preparation protocols of thin-film hydration (for giant assemblies) and ethanol injection (for small assemblies), following the adapted procedures described by Percec et al. [3] and García-Manrique et al. [35]. Thin-film hydration method consists in the hydration of stacked dendrimer bilayers that separate and self-close, forming the assemblies [40]. The ethanol injection method is performed by the injection of an ethanol solution of the dendrimers through a thin needle into an aqueous solution while stirring [41]. Assemblies are formed instantaneously [42], with particle sizes smaller than the obtained by thin-film hydration method.

#### 4.4.1. Giant Assemblies

A 10 mg/mL solution of dendrimer in chloroform (200 µL) and a 3 mg/mL solution of Nile Red (10 µL) in the same solvent were mixed and deposited on a 2 cm^2^ roughened Teflon plate. After solvent evaporation, the Teflon plate was placed in a vial and continued drying under reduced pressure for 2 h. Addition of 4 mL ultrapure water and subsequent hydration at 65 °C for 1 h followed by hydration at 23 °C for 12 h, led to the formation of the aggregates. Assemblies were characterized by confocal microscopy using a Leica TCS SP5 confocal microscope (Leica Microsystems, Wetzlar, Germany), equipped with an HCX PL APO CS 20.0 × 0.70 IMM UV objective (Leica Microsystems, Wetzlar, Germany). Nile Red was excited at 488 with an argon laser and emission spectra collected at 600–700 nm. Confocal images of 512 × 512 pixels were acquired in the XYZ scan mode at a scan speed of 400 Hz. Optical sections were taken at 1.98 µm intervals. Images were digitalized at a resolution of 8 bits. The digital images of the confocal stacks were processed using Fiji software (ImageJ, National Institutes of Health, Bethesda, MD, USA) [43].

#### 4.4.2. Small Assemblies

A dendrimer solution (10 mg/mL, 100 µL) in absolute ethanol was injected into ultrapure water (1.9 mL) and vortex mixed for 5 s, to obtain a final dendrimer concentration of 0.5 mg/mL. The size, PDI, and ζ-potential of the assemblies were determined by triplicate (independent samples from each treatment) at 23 °C, using dynamic light scattering (DLS) with a Malvern Zetasizer ZS ZEN3600 (Malvern Instruments Ltd., Malvern, UK) following the procedure described by Percec et al. [3]. Morphology of the assemblies was evaluated using atomic force microscopy (AFM). For this analysis, samples obtained from ethanol injection experiments were diluted 1:2 with ultrapure water. 10 μL of the sample were placed on a glass coverslip and then allowed to air dry for approximately 15 h. Samples were observed in a NT-MDT NTEGRA Prima AFM (Moscow, Russia) at 23 °C, with a RTESPA probe (Bruker, Billerica, MA, USA) of spring constant k = 40 N/m in semicontact mode. Height and phase images were simultaneously obtained with a scan rate of 1.61 μm/s over a selected area of 1 × 1 μm. Images were processed and analyzed using NOVA 3.1. (NT-MDT). The height and diameter of assemblies were measured from the profile section of AFM line scans analyzing height images

### 4.5. Coarse-Grained Molecular Dynamic Simulation

**Figure 8:**
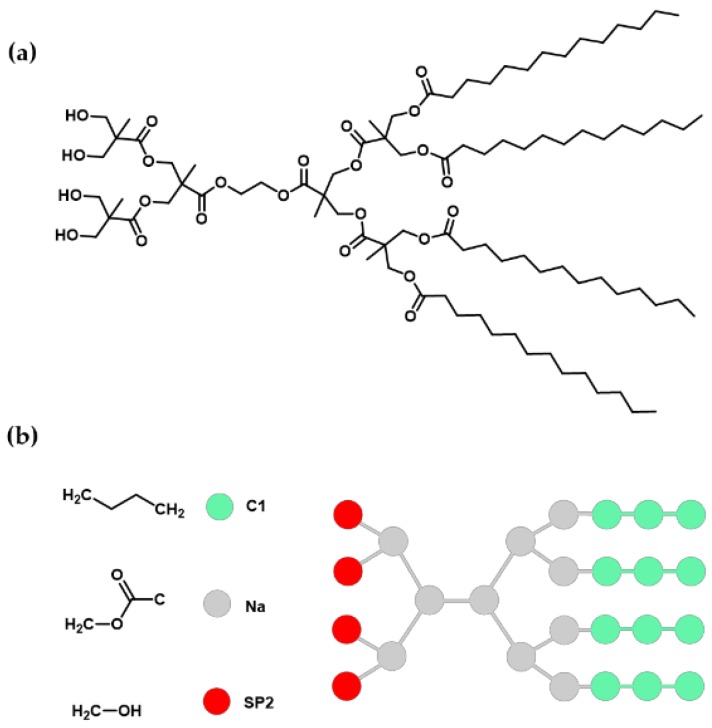
Mapping between the molecular structure and coarse-grained (CG) model for the amphiphilic Janus dendrimer. (**a**) Dendrimer molecular structure (full-atom); (**b**) The simplified structure using CG beads. Bis-MPA methyl substituents were not considered.

CG-MD simulation was made using the MARTINI CG model [44]. The parameters of this model (available at http://www.cgmartini.nl/images/parameters/ITP/martini_v2.0_polymers.itp) were adapted to use with the dendrimer composed of 2 G2 bis-MPA dendrons with 4 OH terminal groups and 4 C_13_ alkyl chains. The simplification of this structure to CG model resulted in the use of three types of beads: three C1 beads and a Na bead for each myristoyl group, six Na beads corresponding to the methyl formate blocks, and four SP2 beads for the hydroxymethyl groups. Bis-MPA methyl substituents were not considered. The dendrimer structure and its bead mapping are shown in Figure 8.

Angle and bond parameters for CG model were obtained from a full atomistic simulation of a single molecule of dendrimer in explicit water and transformed to CG resolution using the mapping technique described previously and following the procedure described by Marquez-Miranda et al. [15], and following Marrink et al. [45,46] criteria to obtain parameters for known bead types. Furthermore, radii of gyration for the full atomistic model was shown similar to CG model.

Simulation system was built with 1700 CG-dendrimers placed randomly into a 37.6 nm × 37.4 nm × 37 nm non-polarized MARTINI water box, representing a dendrimer concentration of 76 mM.

The CG simulation was performed using GROMACS simulation package 5.0.3 (SciLifeLab, Stockholm, Sweden). Steepest descent method was used for energy minimization with a force tolerance of 10 kJ mol^−1^ nm^−1^. After, a molecular dynamics simulation in the isobaric-isothermal ensemble was performed under periodic boundary conditions with a temperature of 310 K and pressure of 1 bar. The temperature was maintained by the velocity rescaling thermostat (modifies Berendsen) [47] and pressure by the Parrinello-Rahman scheme. Lennard-Jones potentials and short-range electrostatics were shifted from 0.9 and 0.0 nm, respectively, to the cut-off distance (1.2 nm) using the standard shift function in GROMACS [48]. Long-range electrostatics were calculated using particle mesh Ewald summation [49]. An integration time step of 30 fs and Verlet algorithm were considered. The total simulation length was 13 μs. Analysis and visualization of simulation results were performed using Tcl homemade-scripts, VMD 1.9.3 (University of Illinois, Urbana, IL, USA) [50] and GROMACS programs. 

### 4.6. Statistics

Data were expressed as mean ± standard deviation. Mean differences were evaluated by unpaired Student’s *t*-test, considering significant a *p*-value < 0.05. Statistical analysis was performed with MINITAB 15 (Minitab Inc., Champaign, IL, USA).

## 5. Conclusions

The self-assembly behavior of a second-generation bis-MPA amphiphilic Janus dendrimer in water was evaluated using experimental methods and CG-MD simulation. Dendrimer structure favors the spontaneous formation of bilayered assemblies in water without interdigitated components, as confirmed by the experimental and simulation results. Assemblies with dendrimersome morphology were obtained by the ethanol injection method. In addition, the CG-MD simulation allowed us to gain a molecular insight into the mechanism of self-assembly of the dendrimer, in which disk-like bilayers were important intermediates of the assembly obtained at the end of the simulation. This is the first report where a CG model for the studied bis-MPA dendrimer is developed and its self-assembly phenomenon is explored. The information presented here is relevant for the design of novel amphiphilic Janus dendrimers with assemblies adapted to be applied to drug delivery systems. For further research, a computational and experimental study of dendrimer structure modifications and their impact in the self-assembly process is in progress.

## Supplementary Materials

The following are available online, Figure S1: Time stability of small assemblies from JD, Figure S2: Temperature stability of small assemblies from JD, Figure S3: Solvent accessible surface area (SASA) of the dendrimers during the simulation, Figure S4: Reaction scheme, Figure S5: ^1^H NMR spectra (500 MHz, CDCl_3_) of JD and peak assignments.
