# Supplementary material for: Self-Assembly Behavior of Amphiphilic Janus Dendrimers in Water: A Combined Experimental and Coarse-Grained Molecular Dynamics Simulation Approach
Source: Molecules. 2018 Apr 21;23(4):969. doi: 10.3390/molecules23040969 (PMC6017225; doi:10.3390/molecules23040969)
Supplement: Supplementary file 1 [file molecules-23-00969-s001.pdf]

Supplementary Material

Self-assembly Behavior of Amphiphilic Janus Dendrimers in Water: A Combined Experimental and Coarse-Grained Molecular Dynamics Simulation Approach

Mariana E. Elizondo-García [1,*], Valeria Márquez-Miranda [2], Ingrid Araya-Durán [2], Jesús A. Valencia-Gallegos [1,*] and Fernando D. González-Nilo [2,3]

1 Escuela de Ingeniería y Ciencias, Tecnológico de Monterrey, Av. Eugenio Garza Sada 2501 Sur, Monterrey 64849, Mexico
2 Center for Bioinformatics and Integrative Biology (CBIB), Facultad de Ciencias Biológicas, Universidad Andrés Bello, Av. República 330, Santiago 8370186, Chile; valeria.marquez.m@gmail.com (V.M.M.); ingrid.araya.duran@gmail.com (I.A.D.); fernando.gonzalez@unab.cl (F.D.G.N.).
3 Centro Interdisciplinario de Neurociencia de Valparaíso, Facultad de Ciencias, Universidad de Valparaíso, Gran Bretaña 1111, Playa Ancha, Valparaíso 2360102, Chile
* Correspondence: mariana.elizndo@gmail.com (M.E.E.G); valencia@itesm.mx (J.A.V.G); Tel.: +52-818-358-2000 (ext. 4511) (J.A.V.G)

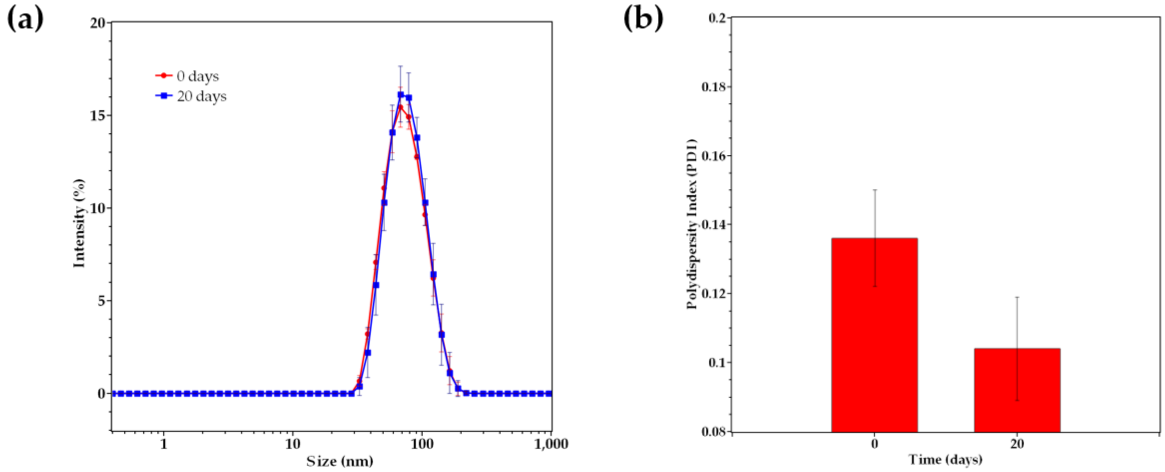

Time	Z-average	PDI
0 days	69.6 ± 1.92	0.14 ± 0.01
20 days	70.8 ± 4.07	0.10 ± 0.01

Figure S1. Time stability of small assemblies from JD. (a) Size distribution by intensity and (b) PDI variation (by DLS).

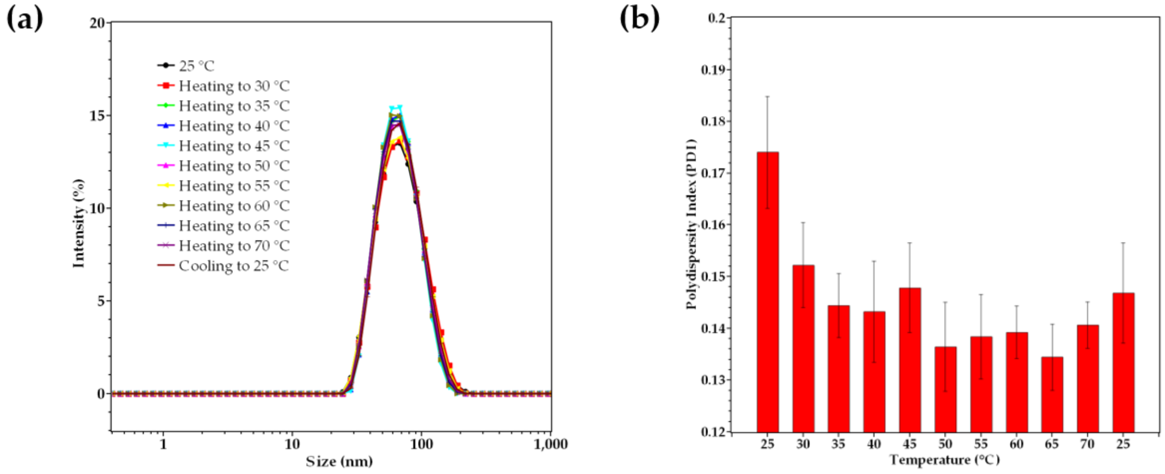

Figure S2. Temperature stability of small assemblies from JD. (a) Size distribution by intensity and (b) PDI variation (by DLS).

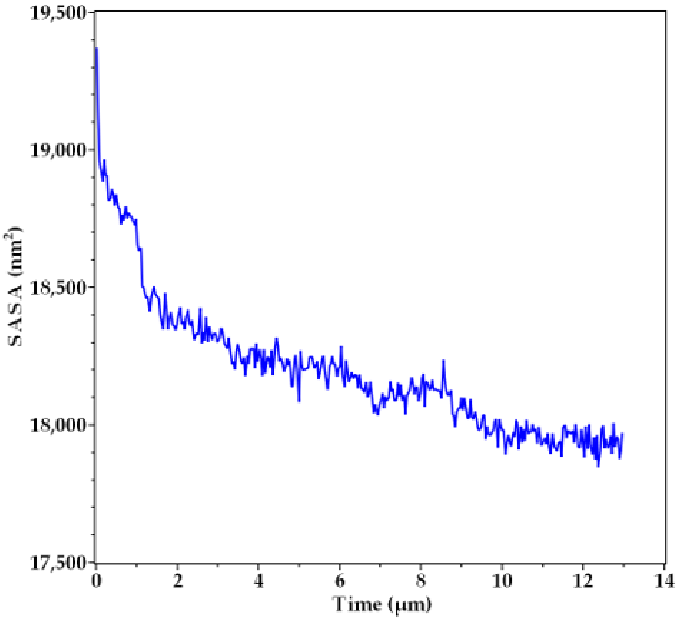

Figure S3. Solvent accessible surface area (SASA) of the dendrimers during the simulation.

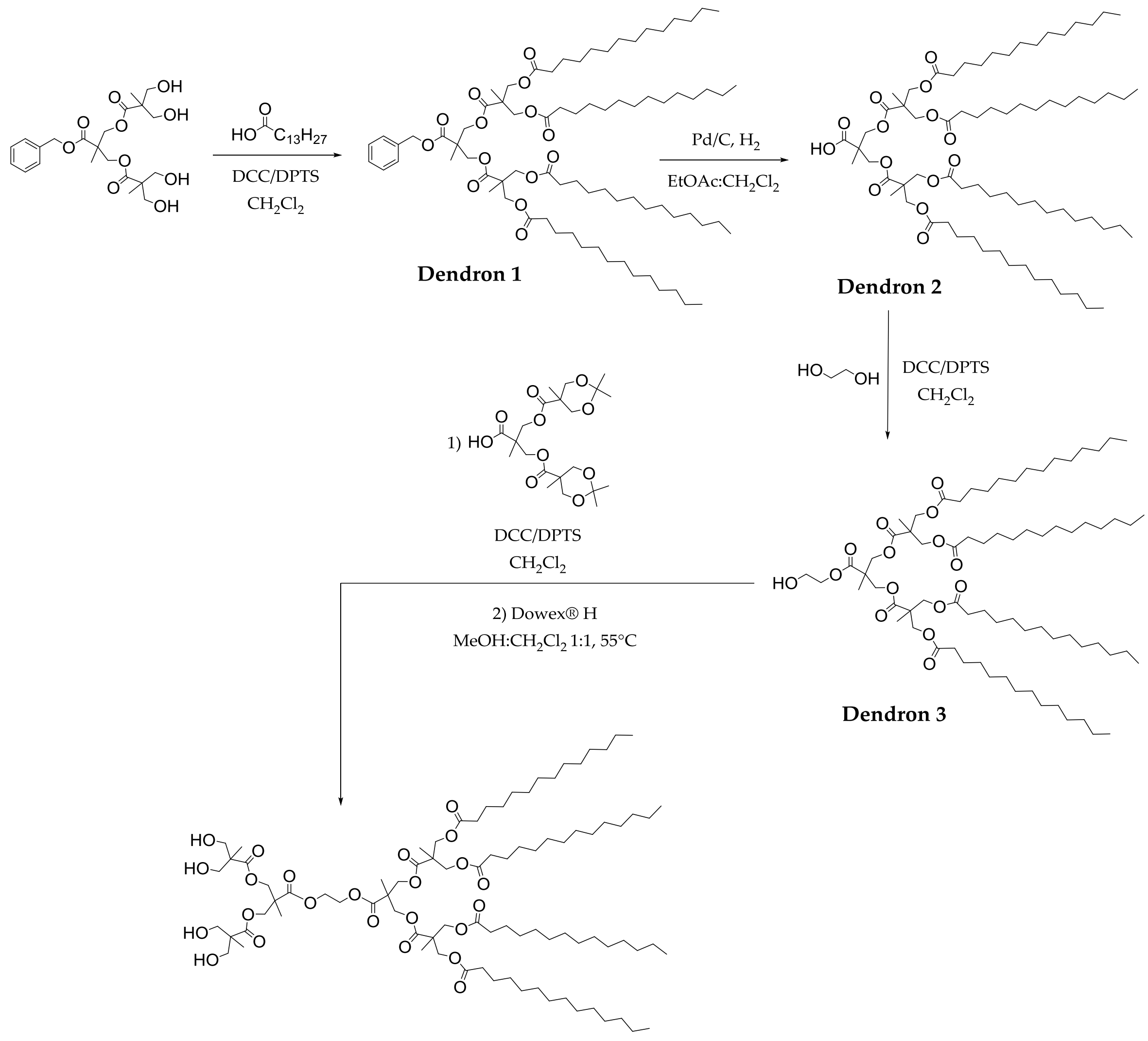

Amphiphilic Janus dendrimer (JD)

Figure S4. Reaction scheme.

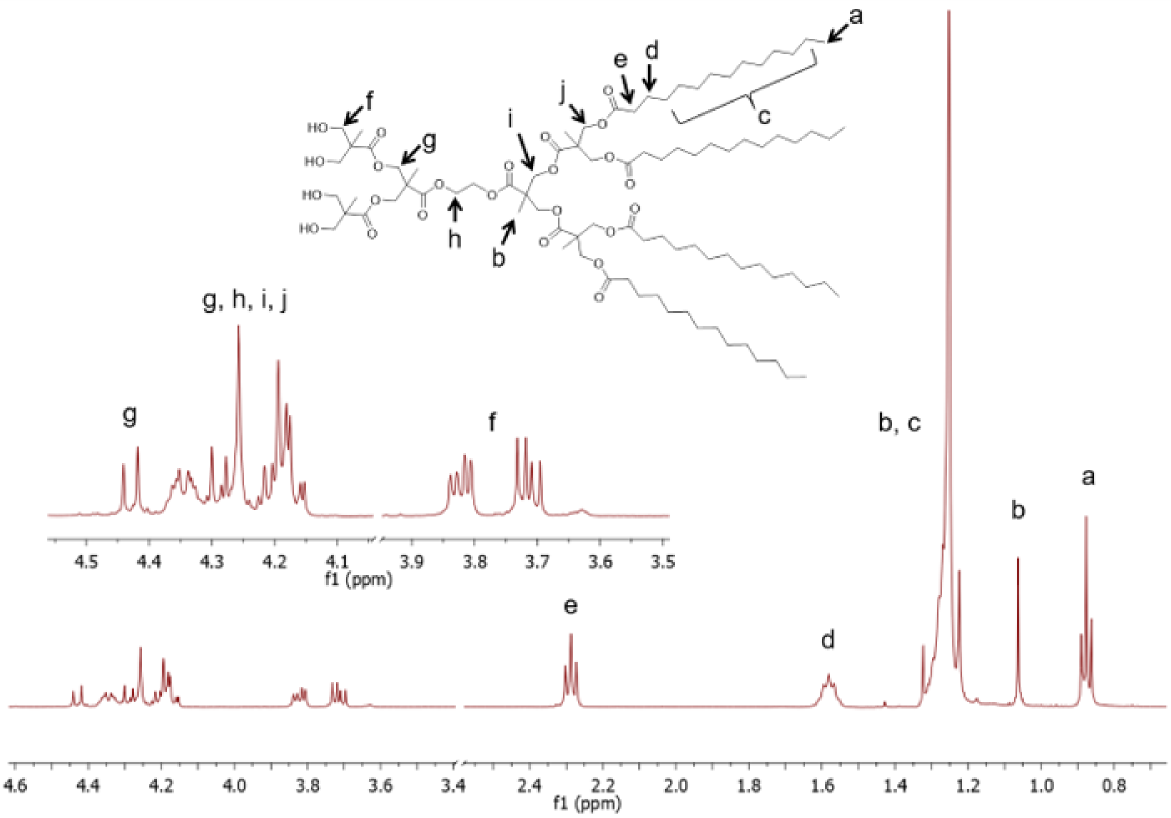

Figure S5. ^{1}H-NMR spectra (500 MHz, $CDCl_3$) of JD and peak assignments.
